# Chemical characterization of wound ointment (WO) and its effects on fracture repair: a rabbit model

**DOI:** 10.1186/s13020-017-0152-y

**Published:** 2017-10-30

**Authors:** Zhixue Ou, Qi Cheng, Yueping Chen, Tao Chen, Xiangbin Rong, Feipan Long, Xiaoyun Zhang, Qinghua Liang, Zhe Feng

**Affiliations:** 1 Department of Traumatic Orthopedics and Hand Surgery, Ruikang Hospital Affiliated to Guangxi University of Chinese Medicine, Block B, No 10 Huadong Road, Nanning, 530011 Guangxi China; 2 Department of Traumatic Orthopedics, Daye Hospital of Traditional Chinese Medicine, Daye, 435100 Hubei China

**Keywords:** Animals, Osteoclasts, Fracture healing, Wound ointment (WO), Radius fracture, VEGF

## Abstract

**Background:**

Wound ointment (WO), a kind of Chinese medicine, can significantly promote fracture healing. The study aimed at analyzing the chemical composition and the effects of WO on fracture of rabbits and tried to explore the corresponding molecular mechanism in cytokine.

**Methods:**

The qualitative and quantitative analysis of WO was conducted by liquid chromatography-mass spectrometry (LC–MS). Fifty-four Zealand mature male rabbits were randomly divided into 3 groups: Control group, Yunnan Baiyao (YB) group and WO group. All the rabbits suffered a fracture of right radius and were then stabilized with an external fixator. Treated with different methods, fracture healing was observed. The bone specimens were subjected to radiograph, immunohistochemistry (IHC) analysis, hematoxylin–eosin staining (HE), western blot and enzyme linked immunosorbent assay (ELISA).

**Results:**

A total of 12 active compositions were detected by LC–MS. Radiographs showed a considerably better bone healing and remodeling of the fracture in WO group. HE experiments showed that a large number of osteoclasts appeared in the early stage when treated with WO. In immunohistochemistry (IHC), western blot and ELISA test, significant increases in vascular endothelial growth factor (VEGF) expression were observed in WO group compared with other two groups.

**Conclusions:**

Wound ointment contained active compositions which efficiently promoted fracture healing through increasing the expression of VEGF.

*Trial Registration* Not applicable

**Electronic supplementary material:**

The online version of this article (doi:10.1186/s13020-017-0152-y) contains supplementary material, which is available to authorized users.

## Background

Bone fracture is a very common disease, which is usually caused by high force, stress, or a minimal trauma injury. An average fracture incidence of 1.17% in males and 1.07% in females has been reported in UK and over 6 million cases occurred in the United States per year [1–4]. Fracture healing is a complicated process, including granulation tissue formation, cartilage callus formation, lamellar bone deposition and remodeling to original bone contour. Surgical management is the most common treatment for fractures, while there is still a risk of morbidity and mortality among the elderly population [5]. Thus, finding a safer and more efficient treatment for fracture is essential. Vascular endothelial growth factor (VEGF) is a sub-family member of growth factors which are important to both vasculogenesis and angiogenesis process. In addition, it has been widely accepted that VEGF played a vital role in bone regeneration through accelerating the process of angiogenesis, which has been confirmed through several animal models as well [6, 7]. There are sufficient evidences supporting that the high expression of VEGF protein can accelerate the healing of femoral fractures in mice and remedy radius segmental defects in rabbits [8, 9].

Yunnan Baiyao (YNBY) is one of the most popular prescriptions of Chinese herbal medicine which possesses haemostatic functions of both open and closed wounds. It is deemed as “a must for first aid” therapy in China of both internal and external hemorrhage as well as open and closed wounds due to its renowned haemostatic functions. It was also reported that YNBY can effectively reduce the severity of murine experimental colitis by both immune-suppressing and wound-healing mechanisms [10–12].

Wound ointment (WO) is made from Chinese medicinal herbs, containing panax, notoginseng, drynaria fortunei and rhubarb etc. Those herbal remedies have active compositions with synergism to relieve pain, prevent infection, and promote healing with less side effects. Antibacterial constituents and analgesic were added in several WOs to bitterly treat wounds and prevent infection. Some uncommon ointments enriching vitamins, including vitamin E, vitamin A and vitamin D can promote skin healing from scrapes, cuts and burns.

The main usage of WOs is to treat soft tissue injury, accelerate blood circulation and relieve congestion, swelling and pain. WO also has anti-inflammatory and antibacterial effects with non-stimulation to skin, heart, liver, kidney and other organs. After traditional disinfection, WO may be dressed directly, or painted onto gauze. It was first applied for fracture regeneration by Chinese medicine doctors [13, 14]. With a clinical application for 30 years, WO has its positive effects on the union of bone confirmed, and exerted a certain influence in China and Southeast Asia [15, 16]. However, there was no relative animal experiment or clinical study to evaluate its efficacy on fracture healing systematically, and its functional mechanism remains unknown to the public due to a lack of researches. These factors mentioned above have largely limited the wide application of WO in clinic. Thus, in this research, we utilized rabbits for radius fracture model to investigate the effects of WO on fracture healing and illustrate the potential mechanisms of WO.

## Methods

### Preparation of sample solutions

A total of 4.0 g WO sample was extracted with 40 mL methanol using Soxhlet extractor for 12 h until the complete extraction. Afterwards, the solution was filtered and evaporated. The crude remnants were uniformly mixed with 5 mL methanol for further analysis.

### Analysis of liquid chromatography-mass spectrometry (LC–MS)

LC–MS was performed using Waters Acquity UPLC system (Waters, Milford, MA, USA) coupled with TOF mass spectrometer (Bruker, Bremen, Germany). The results were acquired using Hystar software (Bruker). Acquity UPLC BEH C18 column (2.1 mm × 100 mm, 1.7 μm) served as the stationary phase, 0.1% formic acid in pure water (A) and 0.1% formic acid in acetonitrile (B) with a flow rate of 0.4 mL/min were utilized as the mobile phase. The conditions were as follows: 0–1 min, 5% B; 1–4 min, 20% B; 4–6 min, 25% B; 6–10 min, 40% B; 10–12 min, 60% B; 12–15 min, 100% B; 15–16 min, 40% B; 16–20 min, 5% B. The column temperature was maintained at 40 °C, and the injection volume of each sample was 2 μL.

The mass spectrometer was operated in the negative mode, the scanning range of *m/z* from 100 to 3000. The mass conditions were as follows: gas (N2) flow rate was 8 L/min and the temperature was 180 °C; the capillary voltage was 4500 V.

### Animal model and fracture surgery

Fifty-Four New Zealand white male rabbits (provided by Animal Center of Guangxi University of Chinese Medicine, Guangxi, China), which were 10 months old with body weight ranging from 2.0 to 2.5 kg, were involved in this study. All rabbits were kept singly and fed with a standard laboratory diet and tap water under climate-controlled conditions (25 °C; 55% humidity; 12 h of light alternating with 12 h of darkness). A small patch of skin dermis was excised from the back and the right forelimb of each rabbit. After the injection of anesthetic (30 mg/mL, sodium pentobarbital sodium solution, Sigma-Aldrich, MO, USA) into the vein of the rabbit ear, all the following procedures were performed in disinfection area with rabbits in supine position. A 1.5 cm longitudinal incision was made to expose the middle part of radius. The periosteum was cut and peeled off, and then the radial shaft was exposed. A fracture model was established by bone sheared with peripheral vessel, muscle and cartilage tissue preserved, keeping the ulna integrate. The research was approved by the ethical committee of Ruikang Hospital Affiliated to Guangxi University of Chinese Medicine. The Minimum Standards of Reporting Checklist contained details of the experimental design, statistics and resources used in this study (Additional file 1).

### Grouping and treatment

The rabbits were categorized into three groups (n = 18 each group). Rabbits received normal saline only were in control group, those treated with Yunnan Baiyao (YB, Yunnan Baiyao group Co., Ltd., Yunnan, China) were regarded as YB group, and WO group consisted of rabbits treated with WO (Ruikang Hospital Affiliated to Guangxi University of Chinese Medicine, Guangxi, China). Five milligram/kilogram of YB and WO were externally applied on the fracture three times a day with bandage being put on and dressings were changed every 3 days. The fracture parts of control group were bandaged without any drug applied.

### Preparation and treatment of specimens

The rabbits were killed at different time point (3, 7, 14, 21, 28, 35 days after operation) by being injected 10–15 mL air into the syringes. After the skin of the fracture was cut immediately, the surrounding muscles, tendons and other soft tissues were stripped. Hyperplastic soft tissue, fibrous and bones callus were halved. One part was fixed in 10% neutral formalin solution and prepared for the following HE stain and IHC analysis; other part was wrapped by tin foil and stored at − 80 °C for western blot. The sacrifice of rabbits and the preservation of the specimens were completed within 10 min to prevent protein degradation and cell autolysis rupture.

### Radiography analysis

The procedure of X-ray and micro-CT observation was similar to the previous studies [17, 18]. Briefly, fracture morphology was detected by digital X-ray equipment (Siemens, Chicago, USA). Three-dimensional (3D) images were rendered. A laboratory micro-CT scanner (Scanco Medical AG, Brüttisellen, Switzerland) and the VGStudio MAX software (Dürr, Bietigheim-Bissingen, Germany) were used for the micro-CT examination. The proportion about total void area of all area in transverse section image was calculated, and the fraction of mineralized callus was quantified.

### Alkaline phosphatase (ALP) assay

At each time point, 4 mL ear margin venous blood stewed in the bio-tube for 30 min was centrifugalized at the speed of 2500 r/min. Then, the upper layer of the serum was removed and ALP activity of the lower layer was detected with ALP assay kit (Sigma-Aldrich, St. Louis, MO, USA). The measurement was performed using Beckman LX 20 Analyzer (Beckman Coulter, Inc., CA, USA).

### Hematoxylin–eosin (HE) staining

The bone specimens were fixed in 10% formaldehyde solution for 48 h and decalcified in 15% ethylene diamine tetraacetic acid (EDTA) for 3 weeks. Then, the decalcified samples were embedded in paraffin, cut into 4 mm-thick sections and dehydrated with gradient ethanol. After that, the sections were stained with HE and assessed with the optical microscope.

### Immunohistochemistry

The bone specimen sections, which were dewaxed and rehydrated with xylene solution and gradient ethanol, were then hot fixed with 0.01 M Citrate repair solution (pH 6.0) for 2 min in hyperbaric condition. 100 μL anti-VEGF receptor 1 (rabbit, 1: 800, ABcam, US) was added and incubated overnight. Then the sections were incubated with horseradish enzyme labeled goat anti-rabbit IgG (Beijing Zhong Jin Jinqiao company, Biological Technology Co., Ltd., Beijing, China) for 40 min. Afterwards, the sections were treated with 3,3′-diaminobenzidine (DAB) coloring agent for 20 s, hematoxylin re-infection for 1 min and dehydrated. The figures were analyzed with the MIQAS medical image quantitative analysis system (Motic China Group Co., Ltd., Guangdong, China), 5 fields of vision were selected randomly with about 100 cells in each field. In each field, mean optical density, the positive reaction area and total area of cells were measured (cells area was 0.15 mm^2^). Subsequently, the average rate of positive area was calculated.

### Western blot

The harvested bone specimens were cut into pieces, weighed and grinded into powder in liquid nitrogen with radio-immunoprecipitation assay (RIPA) buffer. The total proteins were extracted using protein extraction kit (Millipore, Billerica, MA, USA). Equal weight of proteins were loaded and separated by sodium dodecyl sulfate-polyacrylamide gel electrophoresis (SDS-PAGE), transferred to polyvinylidene fluoride (PVDF) membranes and then blocked with 5% skim milk at room temperature for 1 h. Primary antibody anti-rabbit F-box protein 32 (FBXO32) diluted with Tris buffered saline solution (TBST, 1: 2500) was added to the membranes and incubated at 4 °C overnight. The membranes were then incubated with goat monoclonal anti-rabbit, the secondary antibody marked with horseradish peroxidase (HRP, 1: 2000), for 2 h at room temperature. Glyceraldehyde-3-phosphate dehydrogenase (GAPDH) served as the internal control. Signal detection was performed using Chemiluminescence reagent kit (Amersham, IL, USA). The antibodies mentioned above were all bought from Beijing Zhong Shan Jinqiao Biological Technology Co., Ltd. (Beijing, China).

### Enzyme linked immunosorbent assay (ELISA)

The bone specimens were cut off and rinsed with pre-cooled phosphate buffered solution (PBS) (0.01 M, pH = 7.4) to remove residual blood and weighted. The specific volume of PBS (1:9 w/w) was added to a glass homogenizer and sufficiently grinded on ice. After 5000*g* homogenate were ultrasonically broken and thawed, it was centrifuged for 10 min and the supernatant was collected. Rabbit VEGF specific enzyme-linked immunoassay kit (TWp027731, TongWei, Shanghai, China) was used to detect the VEGF expression level. Manufacturer’s protocol was strictly followed and the optical density (OD) was measured at 450 nm.

### Statistical analysis

All statistical analyses were performed by SPSS 19.0 software (SPSS, IL, USA). The significant differences in numerical data (mean ± SD) were estimated with the analysis of variance (ANOVA). Differences between two groups were analyzed by unpaired *t* tests. *P* value less than 0.05 was defined as statistical significance.

## Results

### 12 compositions were detected in WO

As shown in Table 1, a total of 12 compositions were detected in WO. The structures of the active compounds were shown in Additional file 2: Figure S1. Most of compositions have the property of antioxidant, anti-inflammatory and antibacterial. Some compositions have been verified to play a crucial role in the fracture healing, especially asperosaponin VI, which could accelerate the proliferation of the bone marrow mesenchymal stem cells and their differentiation toward osteoblasts [19].

**Table 1:** Comprehensive analysis of WO by LC–MS

Retention time (min)	*m/z*	Mol. wt	Formula	Compounds
2.23	137	154.12	C_7_H_5_O_3_	Protocatechuic acid
2.81	289	308.28	C_15_H_14_O_6_	Catechin
2.86	137	138.12	C_7_H_6_O_3_	p-Hydroxybenzoic acid
4.55	595	596.53	C_27_H_32_O_15_	Neoeriocitrin
5.27	579	580.53	C_27_H_32_O_14_	Naringin
7.98	269	270.23	C_15_H_10_O_5_	Emodin
9.36	928	929.1	C_47_H_76_O_18_	Asperosaponin VI
10.02	266	283.21	C_15_H_7_O_6_	Rhein
10.32	271	272.25	C_15_H_12_O_5_	Naringenin
11.4	285	286.23	C_15_H_10_O_6_	Kaempferol
12.63	283	284.27	C_16_H_12_O_5_	Physcion
13.29	861	862.75	C_42_H_38_O_20_	Sennoside A

### Fracture healing was the fastest in WO group

**Fig. 1:**
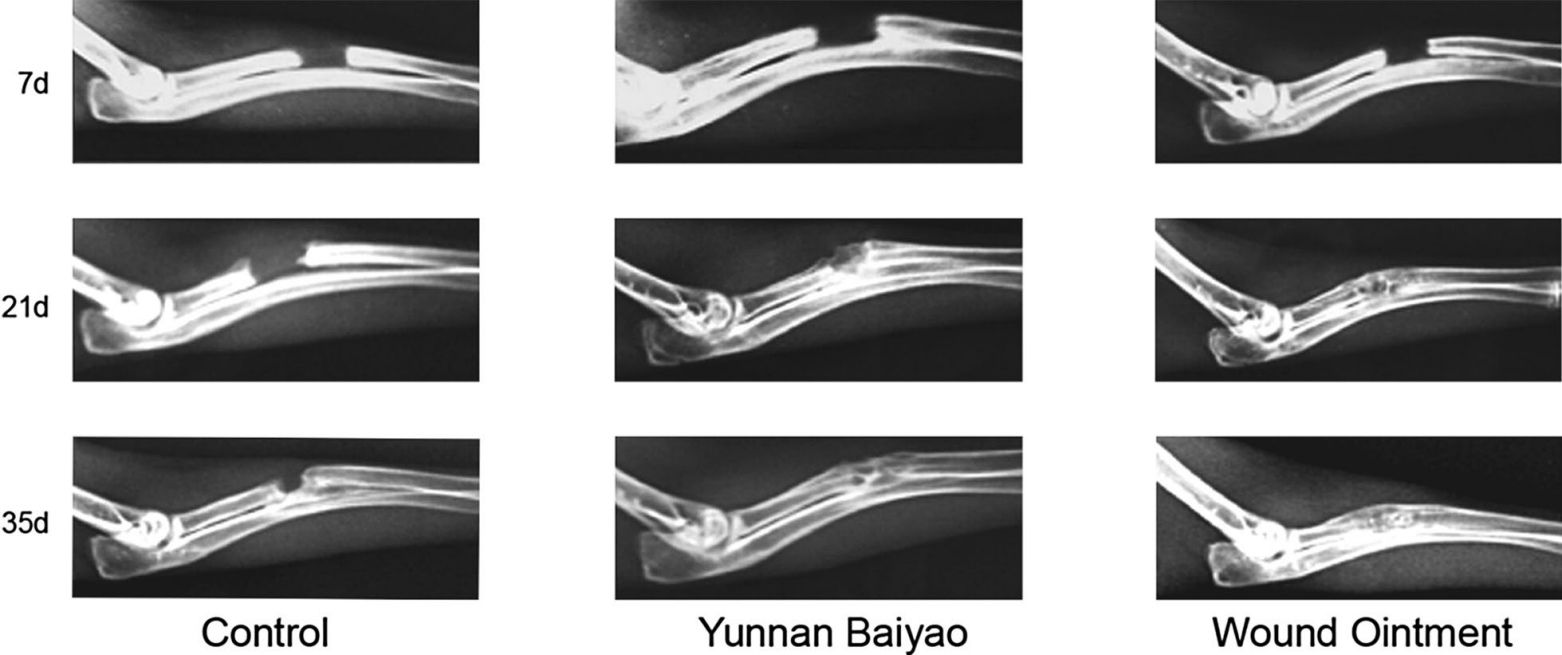
Fracture healing was the fastest in WO group detected by X-ray. X-ray observation of fracture healing conditions at 7, 21 and 35 days after fracture in the control group, Yunnan Baiyao (YB) group and Wound Ointment (WO) group. WO group turned out to be the fastest recovery of fracture

**Fig. 2:**
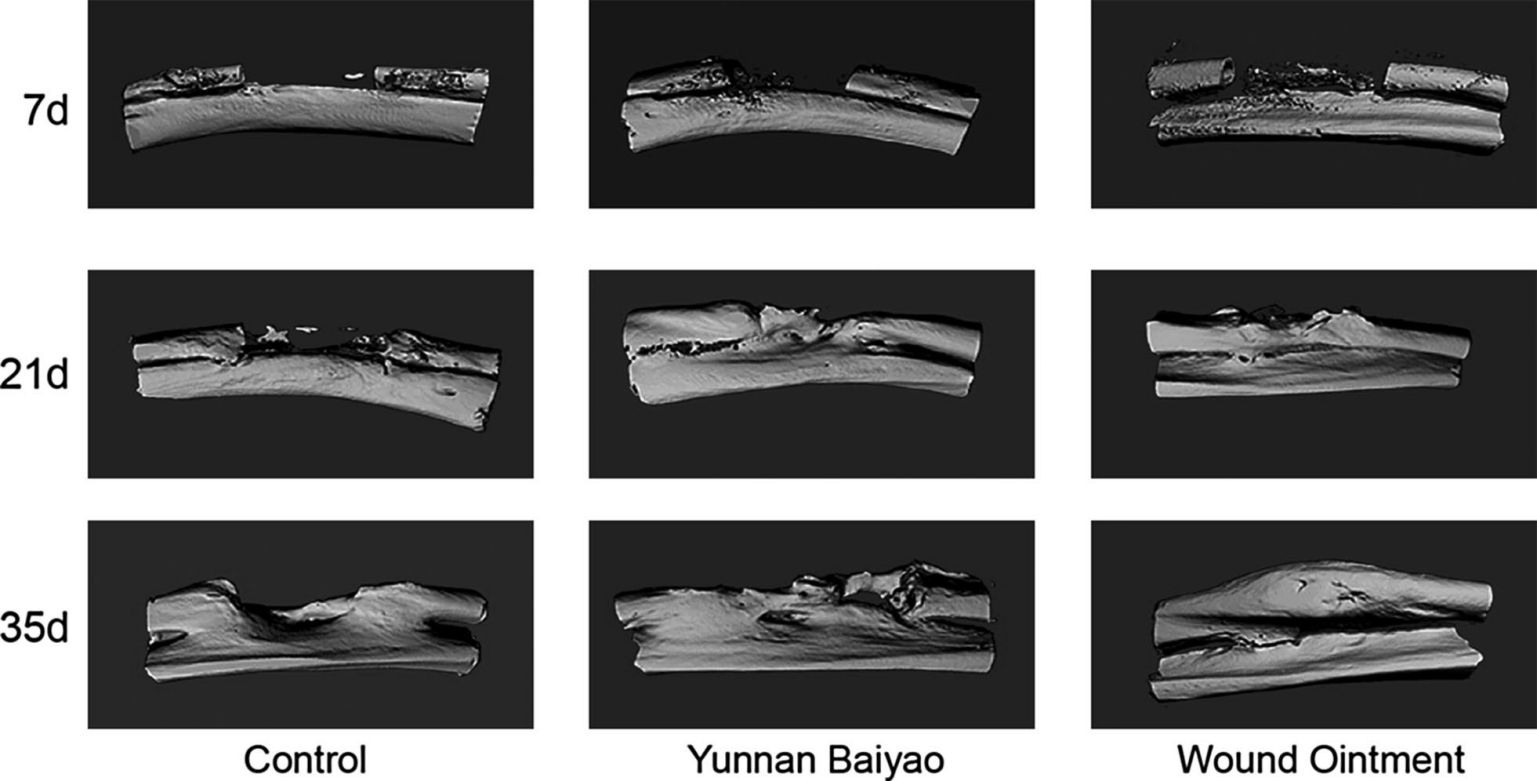
Fracture healing was the fastest in WO group detected by Micro-CT. Micro-CT observation of fracture healing conditions at 7, 21 and 35 days after fracture in the control group, Yunnan Baiyao (YB) group and Wound Ointment (WO) group. Recovery of WO group was the best among all the three groups

The control group showed the common process of fracture healing (Fig. 1), where the fracture line was still clearly observed 21 days after the operation. At the 35th day, the fracture line became blurry, and the bone callus formed. While in YB group, the speed of fracture healing was much faster than that of control group. At the 21st day, the fracture line was fuzzy with massive callus appeared at the fracture end. At the last observation in day 35, the fracture line was almost disappeared, but fracture ghosting still existed. In WO group, the recovery rate of fractured bone was faster than that of other two groups. At the 21st day, the obscure space was very narrow with massive bone callus proliferated. At the 35th day, the fracture line disappeared and the fractured limbed was almost repaired. As shown in Fig. 2, micro-CT results further verified the healing progress and presented more distinct fracture healing condition. The YB group and WO group were much superior to that of control group. In the WO group, the fracture was almost recovered at the 35th day. As bone volume shown in Table 2, the volume of YB and WO group had significant difference compared with that of control group. WO group also showed larger volume in YB group in the 21st day. The results showed that WO and YB could promote the fracture healing in a short time, and the WO had the better effect during the interim period.

**Table 2:** The bone volume of uCT in 7, 21 and 35 days

	Control	YB	WO
7 days	0.479 ± 0.026	0.627 ± 0.047**	0.855 ± 0.057***
21 days	0.684 ± 0.043	0.942 ± 0.061***	1.197 ± 0.066***^###^
35 days	0.875 ± 0.056	1.225 ± 0.073***	1.301 ± 0.105***

** *P* < 0.01, *** *P* < 0.001. There was significant difference between the control group and two treatment group; ^###^ *P* < 0.001. There was significant difference between the YB group and WO group

### Tissues grew fastest in WO group

**Fig. 3:**
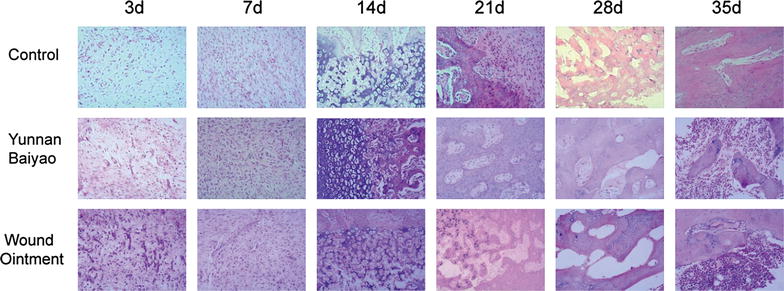
Tissues and VEGF grew fastest in WO group. HE staining of positive signal of vascular endothelial growth factor (VEGF) within the fracture end at each time point (3, 7, 14, 21, 28 and 35 days) in the control group, Yunnan Baiyao (YB) group and Wound Ointment (WO) group. WO group showed the best bone healing effect. *HE* hematoxylin eosin

As shown in Fig. 3, at the 7th day, tissue necrosis and reactive periosteum hyperplasia occurred in control group, with inflammatory cell gathered and capillary proliferated. At the 14th day, little fibrocyte formed with slight reaction while at the 21st day, the proliferation became active and at the 35th day, the bone callus joint with no woven bone molded. As in YB group, there was periosteal reaction and proliferation, and fibroblast gathered obviously at the 7th day. The chondrocyte appeared the 14th day and proliferated at the 21st day with primary callus formed, when subperiosteum ossification, endochondral ossification and primitive trabecula bone could be observed at the fracture end. At the 28th day, fracture was connected with a great deal of woven bones formed. At the last observation, primary marrow cavity appeared, but there was no connection between the upper and lower cavum ossis. Meanwhile, the bone recovery process in WO group was similar to that in YB group during the first 14 days. However, massive bone callus appeared and connected at the 21st day, primary marrow cavity formed at the 28th day and both ends of bone medullary cavity were connected in the last examination. In summary, the WO group showed the best bone healing effect.

### ALP concentration was the highest in WO group

**Fig. 4:**
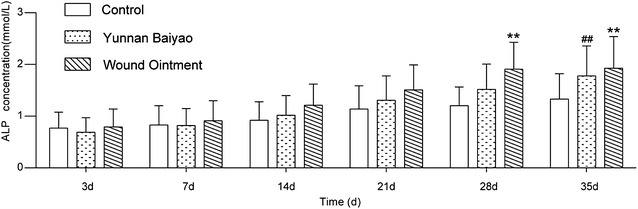
ALP concentration was the highest in WO group. ALP assay detected the value of ALP in fracture tissue at each time point (3, 7, 14, 21, 28 and 35 days) in the control group, Yunnan Baiyao (YB) group and Wound Ointment (WO) group. WO group increased the fastest, suggesting a surprising speed of bone formation. ^##^ *P* < 0.01, YB group versus control group; **P < 0.01, WO group versus control group. *ALP* alkaline phosphatase

ALP activity is an indicator that measures the self-repaired ability. Osteon recovery is strongly associated with the increase of ALP. As shown in Fig. 4, the value of ALP in serum showed an upward tendency in three groups. ALP value in WO group increased significantly compared with the control group (*P* < 0.01) at the 28th day, the relevant phenomena was the surprising speed of bone formation. 35 day after the operation, the ALP level in the YB group and WO group both showed the significant increase compared with the control group (*P* < 0.01).

### WO accelerated fracture healing by promoting the release of VEGF

**Fig. 5:**
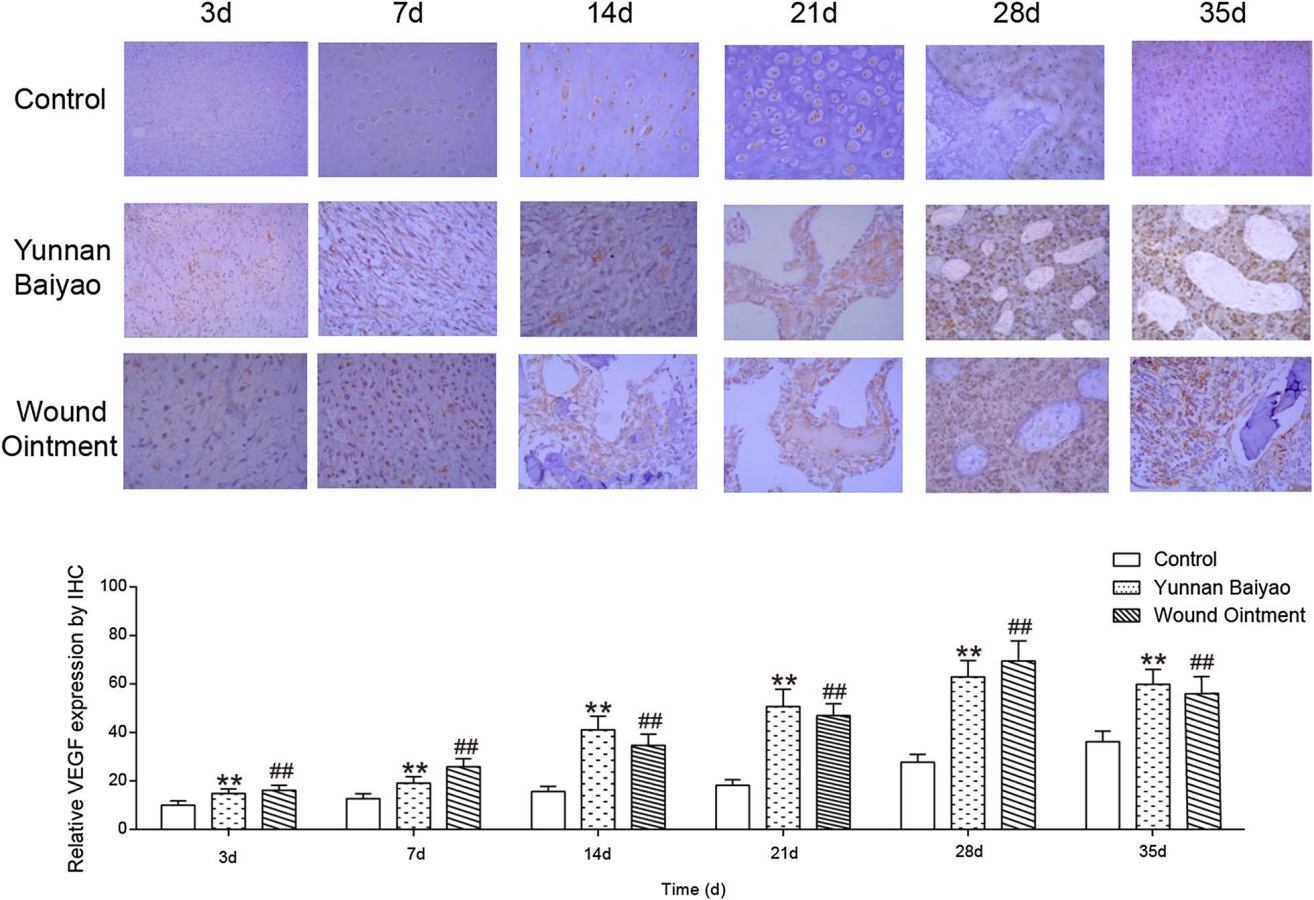
WO accelerated fracture healing by promoting the release of VEGF. Immunohistochemistry of positive signal of vascular endothelial growth factor (VEGF) within the fracture end at each time point (3, 7, 14, 21, 28 and 35 days) in the control group, Yunnan Baiyao (YB) group and Wound Ointment (WO) group. **P < 0.01, YB group versus control group; ^##^ *P* < 0.01, WO group versus control group

The positive substance was located in cytoplasm, in other words, the claybank cytoplasm showed a positive signal (Fig. 5). At the 7th day, in NC group, VEGF expressed high in dense fibrous connective tissue and expressed low in few hyperplastic chondrocytes. The expression of VEGF in YB was stronger than that in control group, and more chondrocytes showed positive VEGF signal. WO group showed the higher expression of VEGF in both the dense connective tissue and the chondrocytes compared with other two groups. In the 14th day, the proliferation of cartilage was active and VEGF was significantly increased while the hypertrophic chondrocytes were less and the contained VEGF was negative. In YB group, the new bone was arranged into trabeculae which was consisted of cartilage and fibrous bones and covered with osteoblasts. These osteoblasts showed positive expression of VEGF signal. Compared with YB group, WO group showed the similar results, while the number of osteoblasts was more and the positive VEGF signal was also stronger. At the 21st day, the typical trabeculae was found and covered a large number of osteoblasts containing VEGF. The number of osteoblasts significantly decreased, however, the size of callus increased in YB and WO group and there was no significant difference between them. After 28 days, there were more callus in control group showed negative expression of VEGF and the number of osteoblasts decreased. In YB and WO group, primary bone marrow cavity appeared and showed slightly positive expression of VGEF, and the expression level was higher in WO than in YB group. At the 35th day, the NC group was in the primary bone marrow cavity stage, YB group and WO group has developed more secondary bone marrow cavity which was relatively more in the WO group and showed higher positive expression of VEGF. The expressions of VEGF in YB group and WO group were much higher than that in control group at each time point (*P* < 0.01), and both reached the peak at the 28th days while the control group increased gradually during the monitoring time. In conclusion, we might speculate that WO promoted the release of VEGF in a short time.

### VEGF was highly expressed in WO group

**Fig. 6:**
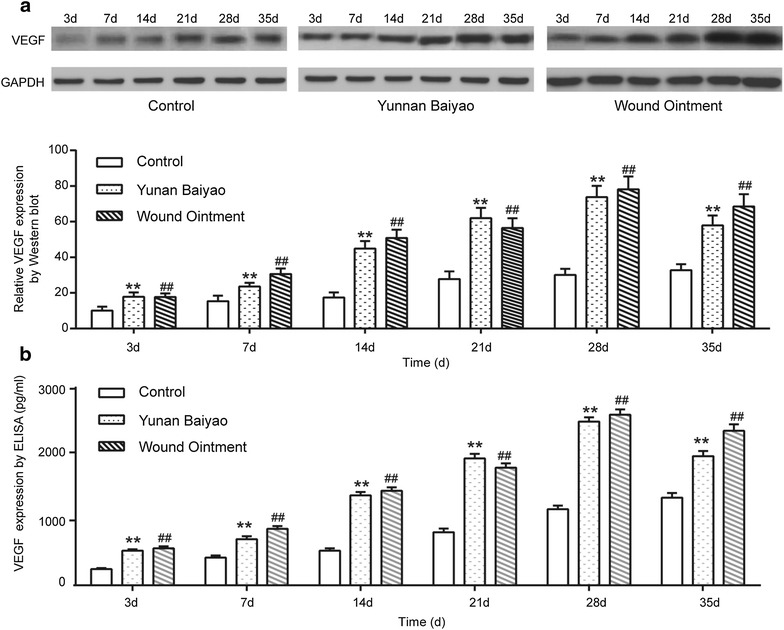
VEGF was higher expressed in WO group. Western blot analysis (**a**) and ELISA (**b**) of VEGF expression within the fracture end at each point (3, 7, 14, 21, 28 and 35 days) in the control group, Yunnan Baiyao (YB) group and Wound Ointment (WO) group. GAPDH was used as loading control. **P < 0.01, YB group versus control group; ^##^ *P* < 0.01, WO group versus control group. *VEGF* vascular endothelial growth factor

Results of western blot further confirmed the regular expression of VEGF protein in the fracture of rabbits during fracture healing in control group, YB group and WO group (Fig. 6a). In the process of fracture healing, the expression level of VEGF in control group was the lowest but increased gradually. After 14 days, the growth was accelerated distinctly. In both YB group and WO group, the expressions of VEGF were augmented dramatically and peaked at the 28th day, and then dropped slightly. Overall, the VEGF expressions in YB and WO group were significantly higher than that in control group (*P* < 0.01). The results were consistent with the IHC analysis.

Results of ELISA confirmed that secretion of VEGF protein in control group, YB group and WO group (Fig. 6b). The trend in the three groups in 35 days was coordinated with that of western blot. The expression of VEGF was higher in the WO group especially in the 28th day, nearly 3000 pg/mL.

## Discussion

A series of physiological processes are involved in bone fracture healing, each of which has fundamental roles in contributing to the healing process [20]. As one of the most important factors in influencing fracture healing, VEGF can stimulate local vascular regeneration in the fracture [21]. There are many studies showing that the application of VEGF can work effectively on angiogenesis and speed the process of bone repair [22–24]. Meanwhile, establishment of animal fracture model will be of great value in the understanding of fracture repair process [25]. The purpose of this research is to investigate whether WO could facilitate the expression of VEGF and accelerate fracture union. Our research was mainly based on a rabbit model of radius fracture. After treatment of Yunnan Baiyao or WO, the healing situation was assessed and compared with the control group. The experimental data showed that WO behaved well in promote neovascularization and callus regeneration via up-regulating the expression of VEGF, which contributed to accelerating fracture healing.

X-ray and micro-CT were logically applied to evaluate the fracture degree, bone strength, as well as fracture healing degree, because of their functions in quantitatively analyzing the microarchitecture of bones [26, 27]. Our observations of X-ray and micro-CT in different time points revealed that in terms of the degree of callus formation and mineralization in fracture healing, WO group came out to be the best-performing one, followed by the YB group, and the control group was the worst.

Results of histomorphometric analysis in the three groups (control, YB and WO group) showed a similar bone formation process. The first change observed by light microscope was the regeneration of chondroblasts and the formation of hyaline cartilage. Loose connective tissue reverted to dense connective tissue gradually and developed into fibrous callus. With the proliferation, differentiation and growth of osteoblasts, fracture callus gradually replaced fibrous callus. At the last stage, osteoclasts became active. Fracture healing was in the phase of remodeling original bone contour. In WO group, the granulation tissue formation, chondroblasts and osteoblasts proliferation, fibrous callus and fracture callus remodeling process occurred at 3 or 7 days after fracture, which was faster than other groups. Significant cartilaginous osteogenesis and faster new bone growth were seen in WO group. These evidences indicated that WO could accelerate new bone growth through promoting cartilaginous osteogenesis. IHC analysis, Western blot and ELISA assay demonstrated that YB and WO played the same role in influencing VEGF expression, while VEGF showed a stronger expression in WO group than in YB group. When compared with control group, both WO and YB groups could improve the expression of VEGF at different stages of fracture repair, which was consistent to the results of HE analysis.

In this research, we investigated the effect of WO on the expression of VEGF during fracture healing in rabbit radius fracture model for the first time. The conclusion conformed to the results of clinical practices. However, there were still some limitations. First, although the positive control of this study, YB is widely utilized to promote fracture healing in China, but it’s unacquainted for other researchers. The mechanisms concerning how YB accelerated fracture healing were also not widely reported. Second, there are some other proteins such as bone morphogenetic protein 2, osteocalcin and type I collagen that also contributing to fracture healing which were not studied in the present research. Whether WO or YB affects expressions of those factors during the treatment still needs other evidences. Moreover, the real conditions of fracture were different from the experimental condition. The causes for bone fractures are very complex and there are great individual differences among patients. Fractures are associated with different complications such as soft tissue damage, massive bleeding, severe pain, infection, visceral injury and even shock. Thus, experiments in animals are not enough and the mechanism of WO on the treatment of fracture needs more studies.

## Conclusions

In conclusion, we had detected the composition of WO and confirmed that its effect on increasing the VEGF expression in the process of bone regeneration was good for neovascularization and callus regeneration, as well as acceleration of fracture healing. This research will deepen our understanding of the function of WO, and will be of value in the development and application of WO.

## Additional files

**Additional file 1.** The minimum standards of reporting checklist.

**Additional file 2: Figure S1.** The structures of the active compounds obtained by LC–MS were shown.

## Abbreviations

ALP: alkaline phosphatase
DAB: diaminobenzidine
EDTA: ethylene diamine tetraacetic acid
FBXO32: F-box protein 32
GAPDH: glyceraldehyde-3-phosphate dehydrogenase
HE: hematoxylin–eosin staining
PVDF: polyvinylidene fluoride
RIPA: radio-immunoprecipitation assay
SDS-PAGE: sodium dodecyl sulfate-polyacrylamide gel electrophoresis
VEGF: vascular endothelial growth factor
WO: wound ointment
YB: Yunnan Baiyao
YNBY: Yunnan Baiyao
LC–MS: liquid chromatograph-mass spectrometer
ELISA: enzyme linked immunosorbent assay
