# Supplementary figures and images for: Chemical characterization of wound ointment (WO) and its effects on fracture repair: a rabbit model
Source: Chin Med. 2017 Oct 30;12:31. doi: 10.1186/s13020-017-0152-y (PMC5661936; doi:10.1186/s13020-017-0152-y)

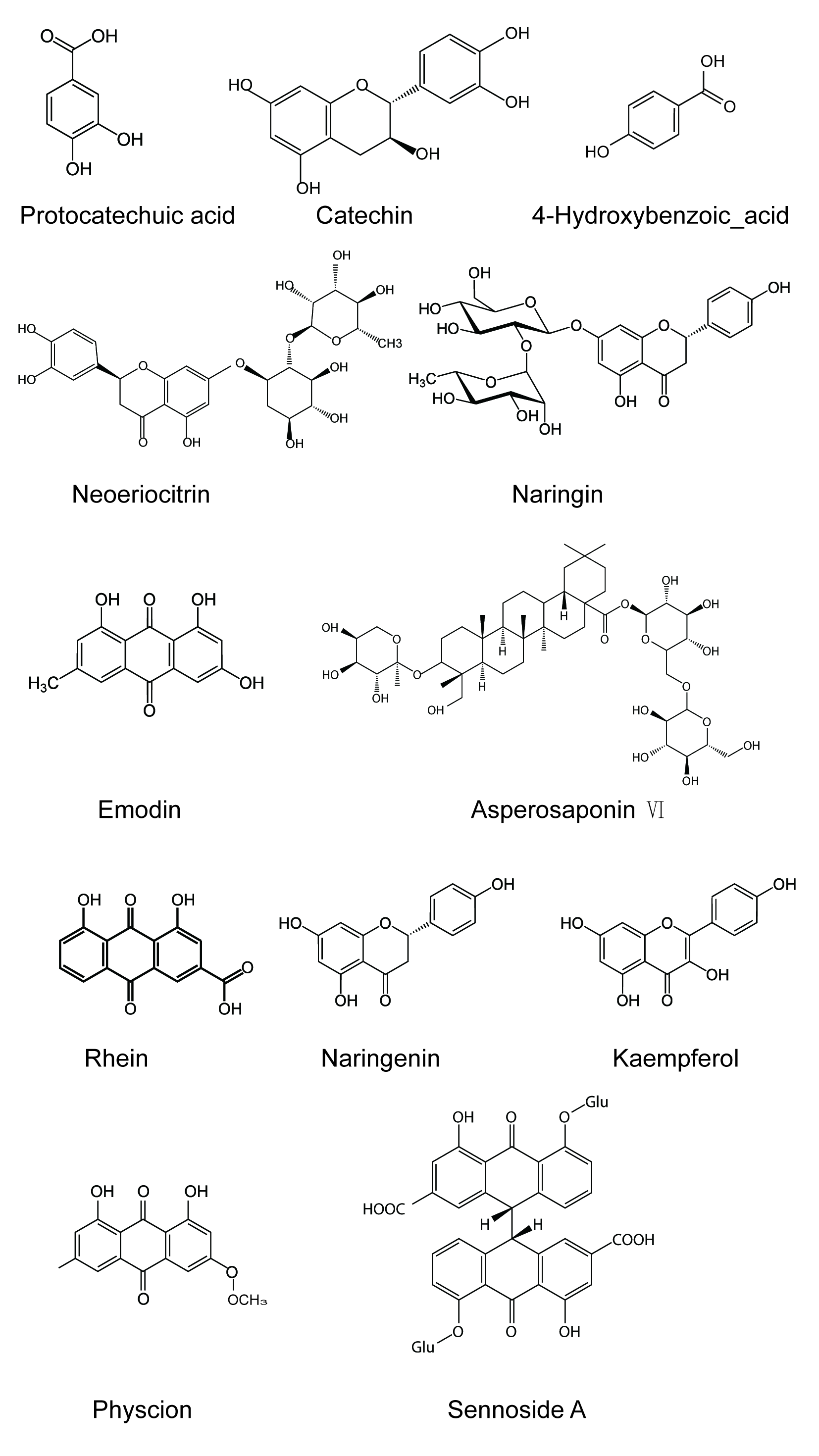

Supplement: Supplementary file 2 — Additional file 2: Figure S1. The structures of the active compounds obtained by LC–MS were shown. [file 13020_2017_152_MOESM2_ESM.tif]
